# Supplementary material for: Global change factors differ in effect when acting alone and in a multi-factor background
Source: Nat Commun. 2026 Jan 9;17:425. doi: 10.1038/s41467-025-68155-9 (PMC12796432; doi:10.1038/s41467-025-68155-9)
Supplement: Supplementary file 1 — Supplementary Information [file 41467_2025_68155_MOESM1_ESM.pdf]

Supplementary Information

Global change factors differ in effect when acting alone and in a multi-factor background

Supplementary note 1

To exclude effects via other parameters, we measured electrical conductivity, ph, phosphorus content and carbon-to-nitrogen-ratio.

pH and electrical conductivity
Soil pH was measured with a pH meter (Knick 761 calimatic, Germany) in a soil-water-solution in a ratio of 1:2,5. Electrical conductivity was measured with a EC meter (SevenEasy pH-Meter Mettler-Toledo, Germany) in a soil-water-solution in a ratio of 1:5.

Phosphorus and Carbon-to-nitrogen-ratio
For C:N determination, the soil was milled and then analyzed with a Euro EA-CN 2 dual elemental analyzer (HEKA Tech, Wegberg, Germany). Soil phosphorus was determined by using a Mehlich-3 extraction solution and absorption spectrophotometry. Soil samples were first homogenized by milling. 100 mg soil were weighed into 1.5 mL microcentrifuge tubes. To each tube, 1 mL of Mehlich-III extraction solution was added, and samples were vortexed for 10 min. Extracts were centrifuged at 8,000 rpm for 3 min, and 450 µL of the supernatant was transferred into 96-well plates. Phosphorus was quantified using the ascorbic acid–molybdate blue method adapted for high-throughput microplate analysis. For matrix matching, 50 µL of Mehlich-III extract was combined with 100 µL of 0.0875 M H_2SO_4 and 50 µL of a freshly prepared mixed reagent containing ammonium molybdate, antimony potassium tartrate, and ascorbic acid. The reaction was allowed to develop for 15–120 min at 37 °C. Absorbance was measured at 850 nm using a microplate reader.Phosphate calibration standards were prepared from a KH_2PO_4 stock solution (50 mg P L^{-1}) serially diluted to concentrations ranging from 0 to 4 mg P L^{-1}. Sample phosphorus concentrations were calculated by fitting absorbance values to the calibration curve.

Supplementary tables and figures

Supplementary table 1: Effect size means (mean_ES) and 95 % confidence intervals (CI_low and CI_high being the lower and upper CI boarder) for all added single treatments compared the control without any treatments (control_0) and the subtracted single factors compared to the combination of all factors (control_all).

Response variable	Factor	comparison	mean_ES	CI_low	CI_high	comparison	mean_ES	CI_low	CI_high
N-acetyl-β-glucosaminidase activity	microplastic	control _0	0.071379	-0.07091	0.221922	control _all	0.052897	-0.02417	0.122976
	warming	control _0	0.229512	0.082673	0.384624	control _all	0.276712	0.168821	0.362766
	salinity	control _0	-0.12896	-0.28836	0.009311	control _all	0.297302	0.207294	0.390074
	Ndeposition	control _0	0.043926	-0.08839	0.185753	control _all	0.061545	-0.02573	0.17733
	drought	control _0	0.085998	-0.07878	0.286887	control _all	0.432305	0.297888	0.545646
	surfactant	control _0	0.051132	-0.08381	0.182551	control _all	0.052828	-0.02549	0.128191
β-D-glucosidase activity	microplastic	control _0	0.797389	0.286838	1.509769	control _all	-0.08094	-0.40075	0.268245
	warming	control _0	0.410842	-0.04832	0.800999	control _all	0.865244	0.463647	1.216507
	salinity	control _0	-0.7757	-1.12492	-0.37575	control _all	0.873755	0.484294	1.308973

	Ndeposition	control _0	0.263554	-0.05497	0.572746	control _all	-0.03262	-0.38216	0.323849
	drought	control _0	0.325325	-0.13777	0.753439	control _all	0.682404	0.311606	1.084747
	surfactant	control _0	0.964169	0.364872	1.933133	control _all	-0.02205	-0.31768	0.268002
	microplastic	control _0	0.214687	-0.99953	1.013566	control _all	-0.08886	-0.49313	0.260217
	warming	control _0	0.316677	-0.94817	1.203303	control _all	2.136252	1.179296	2.877618
Phosphatase activity	salinity	control _0	-2.24254	-3.45494	-1.46688	control _all	2.826023	2.07097	3.643539
	Ndeposition	control _0	-0.22622	-1.56291	0.684691	control _all	-0.17149	-0.66188	0.372901
	drought	control _0	0.684417	-0.69919	1.79278	control _all	1.43001	0.91912	2.002711
	surfactant	control _0	0.599723	-0.57855	1.731583	control _all	-0.12578	-0.63466	0.432743
	microplastic	control _0	-0.00412	-0.05444	0.040303	control _all	-0.03099	-0.1821	0.025945
	warming	control _0	0.165105	0.125023	0.202283	control _all	-0.0681	-0.22608	-0.01347
Decomposition rate	salinity	control _0	-0.16325	-0.1932	-0.13211	control _all	0.469136	0.317331	0.532419
	Ndeposition	control _0	-0.00715	-0.04578	0.037426	control _all	-0.04666	-0.19855	0.008279
	drought	control _0	0.352417	0.251968	0.400836	control _all	0.089548	-0.06816	0.152224
	surfactant	control _0	-0.00922	-0.05373	0.0388	control _all	-0.0342	-0.1903	0.020707
	microplastic	control _0	0.112906	-0.04626	0.275635	control _all	-0.02736	-0.15193	0.022398
	warming	control _0	-0.06667	-0.22034	0.113933	control _all	0.041337	-0.07462	0.108829
Mean weight diameter	salinity	control _0	-0.08391	-0.21136	0.09193	control _all	-0.00538	-0.10661	0.091163
	Ndeposition	control _0	-0.11749	-0.26315	0.067335	control _all	-0.01469	-0.13892	0.041488
	drought	control _0	-0.4943	-0.61237	-0.3325	control _all	0.639926	0.471505	0.841696
	surfactant	control _0	0.060336	-0.1411	0.269271	control _all	0.005676	-0.09925	0.096499
	microplastic	control _0	0.787152	-3.3729	6.988092	control _all	-0.88632	-4.23525	3.461082
	warming	control _0	7.753441	3.38714	13.3497	control _all	1.95871	-2.89224	7.019771
Water-stable aggregates	salinity	control _0	-11.9071	-16.7061	-6.08235	control _all	15.80408	11.42054	20.59449
	Ndeposition	control _0	-3.79204	-9.1346	2.782746	control _all	2.131838	-1.79237	6.733942
	drought	control _0	-4.10549	-9.24637	1.185143	control _all	5.94962	-0.01692	11.89804
	surfactant	control _0	-0.92537	-6.04006	5.847802	control _all	2.064138	-2.05347	7.00963
	microplastic	control _0	-0.35	-0.95	-0.15	control _all	-9.19444	-20.0655	-0.64352
	warming	control _0	-0.35	-0.95	-0.15	control _all	-12.6444	-23.0503	-3.7548
Water drop penetration time	salinity	control _0	-0.3	-0.8	0	control _all	17.55556	5.262812	29.63333
	Ndeposition	control _0	-0.15	-0.7	0.2	control _all	-2.89444	-15.1459	8.358107
	drought	control _0	5.45	3.25	7.8	control _all	-24.9444	-34.5	-17.5
	surfactant	control _0	-0.3	-0.8	0	control _all	10.15556	-3.21177	23.421

Supplementary table 2: Redundancy analysis (RDA) and variance partitioning to quantify the relative contribution of each factor, their interactions, and residual (biological noise or measurement errors) for all- group (independent R²).

Response variable	Factor	Independent R²
N-acetyl-β-glucosaminidase activity	Warming	0.159079
	Drought	0.396307
	Salinity	0.184495
	Surfactant	0.000441
	N deposition	0.002569
	Microplastic	0.000426
	Shared (interaction)	0.000000
	Residual	0.351265
β-D-glucosidase activity	Warming	0.127104
	Drought	0.075372
	Salinity	0.129810
	Surfactant	0.000000
	N deposition	0.000000
	Microplastic	0.000000
	Shared (interaction)	0.052782
	Residual	0.614933
Phosphatase activity	Warming	0.155493
	Drought	0.066334
	Salinity	0.276659
	Surfactant	0.000000
	N deposition	0.000000
	Microplastic	0.000000
	Shared (interaction)	0.120065
	Residual	0.381449
Decomposition	Warming	0.008262
	Drought	0.015494
	Salinity	0.469099
	Surfactant	0.000396
	N deposition	0.002998
	Microplastic	0.000844
	Shared (interaction)	0.398437
	Residual	0.104470
Mean weight diameter	Warming	0.000000
	Drought	0.443133
	Salinity	0.000000
	Surfactant	0.000000
	N deposition	0.000000
	Microplastic	0.000000
	Shared (interaction)	0.287355
	Residual	0.269512
Water-stable aggregates	Warming	0.000000
	Drought	0.036772
	Salinity	0.313569
	Surfactant	0.000000
	N deposition	0.000000
	Microplastic	0.000000
	Shared (interaction)	0.086826
	Residual	0.562834
	Warming	0.031138
	Drought	0.142639

Water drop penetration time	Salinity	0.066906
	Surfactant	0.012967
	N deposition	0.000000
	Microplastic	0.017452
	Shared (interaction)	0.261426
	Residual	0.467471
Electrical conductivity	Warming	0.002019
	Drought	0.000000
	Salinity	0.552363
	Surfactant	0.001124
	N deposition	0.000000
	Microplastic	0.000000
	Shared (interaction)	0.274997
	Residual	0.169497
pH	Warming	0.029258
	Drought	0.086963
	Salinity	0.072416
	Surfactant	0.125286
	N deposition	0.007198
	Microplastic	0.034358
	Shared (interaction)	0.000000
	Residual	0.908833
Phosphorus	Warming	0.0000000
	Drought	0.0000000
	Salinity	0.0000000
	Surfactant	0.0000000
	N deposition	0.0187398
	Microplastic	0.0000000
	Shared (interaction)	0.0000000
	Residual	1.0237610
Carbon-to-nitrogen ratio	Warming	0.0000000
	Drought	0.0000000
	Salinity	0.003527902
	Surfactant	0.0000000
	N deposition	0.0000000
	Microplastic	0.0000000
	Shared (interaction)	0.0000000
	Residual	1.051575794

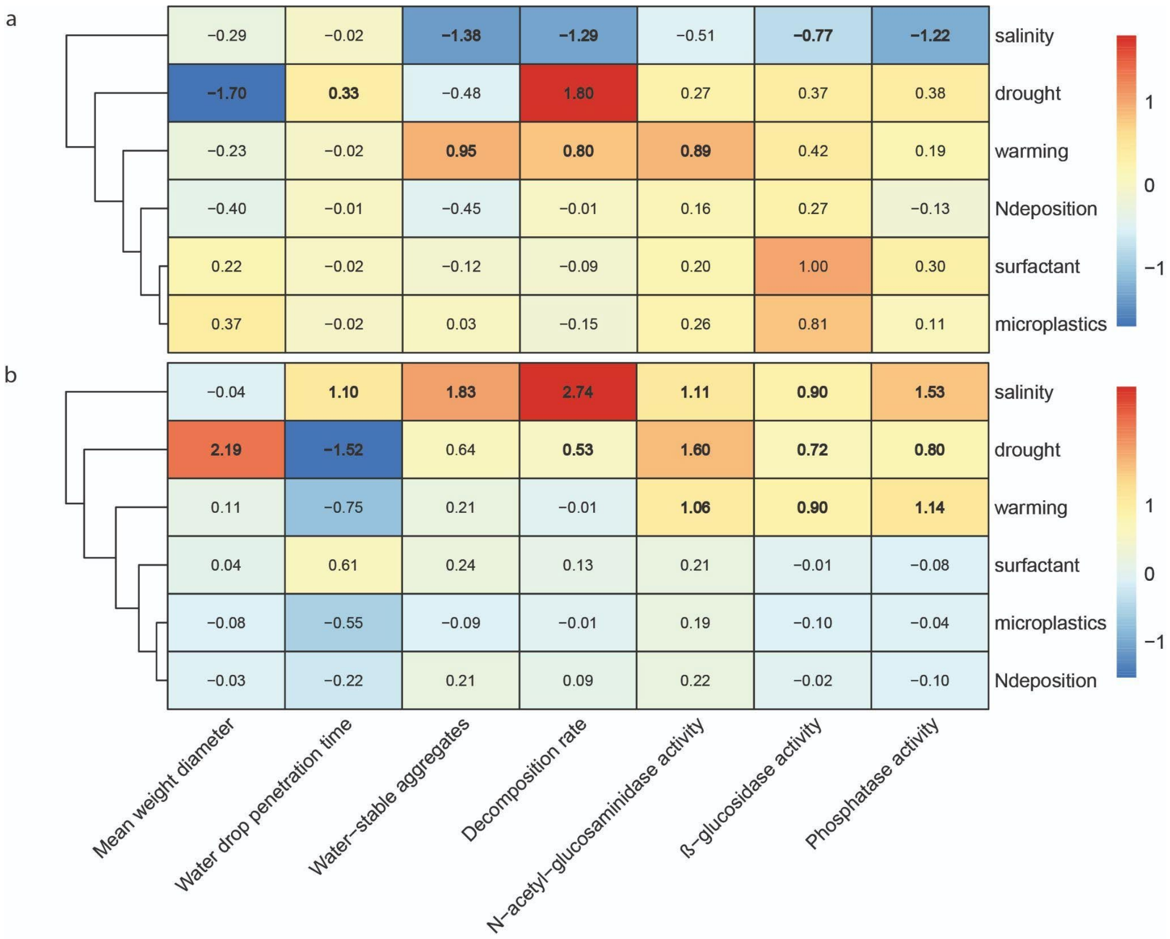

Supplementary figure 1: **Clustered heatmaps of adjusted mean effect size of all tested response variables.** a y-axis is the single factor, mean effect size calculated as the difference between single factor and control. b y-axis is the factor that minused from six factor level, mean effect size calculated as the difference between six factors and five factors. Cluster analysis calculated based on Euclidean distance. Mean effect size adjusted using the z-score method. Significance effects were marked with bold numbers. Colour coding represents positive (red) and negative (blue) effect directions. n=10.

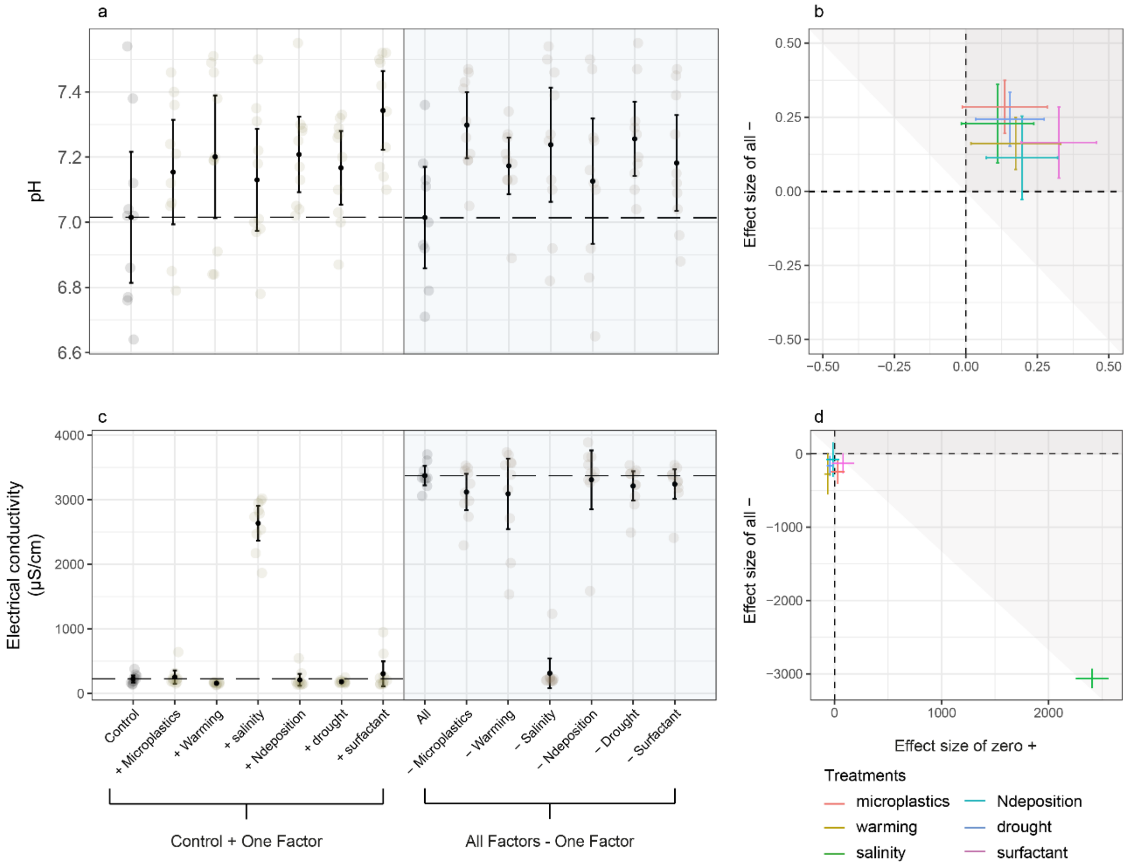

Supplementary figure 2: **pH and electrical conductivity affected by multiple factors of global change**. a, c: The left part of the graph (white background) shows the control and the single factor treatments. The dashed line shows the mean value of the control plots. Dots reflect the measured values (10 replicates each), marked with a black filled dot is the mean, the 95 % confidence interval is shown by the line passing through the dot. On the right side of the graph, the combination of all global change factors (dashed line as mean), is compared to the treatment combinations, from which each of the single factors is subtracted. p values derived from two-sided one-way ANOVA followed by Tukey's post-hoc tests. Redundancy analysis (RDA) and variance partitioning were performed to quantify the relative contribution of each factor, as well as their interactions, and residual (biological noise or measurement errors) c, d: To visualize the effect directions of factor addition and subtraction treatments, bootstrapped (n = 100) effect size of factor addition were plotted on the x-axis and the effect of factor removal on the y-axis. Different factors of global change are represented by different colours. Factor effects on the diagonal red line indicate that the effect of the factor in the multi-factor background has the direction and intensity expected from the single-factor effect. Factor effects in the grey shaded area indicate that a factor in combination with other factors has a more negative effect than expected from the single factor effect. Dark grey areas represent positive single factor effects that have a negative effect when combined with other factors. n=10. Source data are provided as a Source Data file.

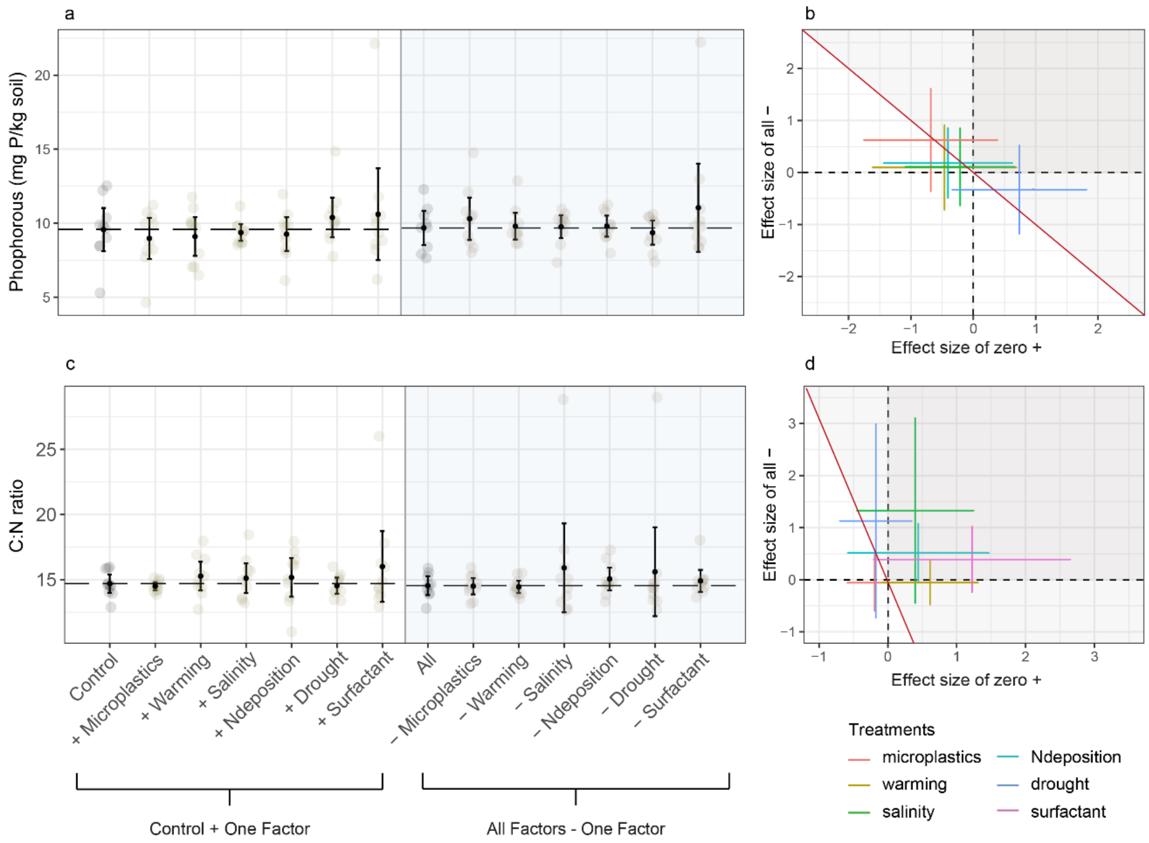

Supplementary figure 3: **Phosphorus and carbon-to-nitrogen-ratio affected by multiple factors of global change**. a, c: The left part of the graph (white background) shows the control and the single factor treatments. The dashed line shows the mean value of the control plots. Dots reflect the measured values (10 replicates each), marked with a black filled dot is the mean, the 95 % confidence interval is shown by the line passing through the dot. On the right side of the graph, the combination of all global change factors (dashed line as mean), is compared to the treatment combinations, from which each of the single factors is subtracted. p values derived from two-sided one-way ANOVA followed by Tukey's post-hoc tests. Redundancy analysis (RDA) and variance partitioning were performed to quantify the relative contribution of each factor, as well as their interactions, and residual (biological noise or measurement errors) c, d: To visualize the effect directions of factor addition and subtraction treatments, bootstrapped (n = 100) effect size of factor addition were plotted on the x-axis and the effect of factor removal on the y-axis. Different factors of global change are represented by different colours. Factor effects on the diagonal red line indicate that the effect of the factor in the multi-factor background has the direction and intensity expected from the single-factor effect. Factor effects in the grey shaded area indicate that a factor in combination with other factors has a more negative effect than expected from the single factor effect. Dark grey areas represent positive single factor effects that have a negative effect when combined with other factors. n=10. Source data are provided as a Source Data file.
